# Lipidomic assessment of the impact of *Nannochloropsis oceanica* microalga lipid extract on human skin keratinocytes exposed to chronic UVB radiation

**DOI:** 10.1038/s41598-023-49827-2

**Published:** 2023-12-15

**Authors:** Wojciech Łuczaj, Agnieszka Gęgotek, Tiago Conde, M. Rosário Domingues, Pedro Domingues, Elżbieta Skrzydlewska

**Affiliations:** 1 Department of Analytical Chemistry, Medical University of Bialystok, Mickiewicza 2D, 15-222 Bialystok, Poland; 2 Mass Spectrometry Centre, LAQV-REQUIMTE, Department of Chemistry, University of Aveiro, Santiago University Campus, 3810-193 Aveiro, Portugal; 3 CESAM-Centre for Environmental and Marine Studies, Department of Chemistry, University of Aveiro, Santiago University Campus, 3810-193 Aveiro, Portugal

**Keywords:** Lipids, Phospholipids, Sphingolipids

## Abstract

Considerable attention has been devoted to investigating the biological activity of microalgal extracts, highlighting their capacity to modulate cellular metabolism. This study aimed to assess the impact of *Nannochloropsis oceanica* lipid extract on the phospholipid profile of human keratinocytes subjected to UVB radiation. The outcomes revealed that treatment of keratinocytes with the lipid extract from microalgae led to a reduction in sphingomyelin (SM) levels, with a more pronounced effect observed in UVB-irradiated cells. Concomitantly, there was a significant upregulation of ceramides CER[NDS] and CER[NS], along with increased sphingomyelinase activity. Pathway analysis further confirmed that SM metabolism was the most significantly affected pathway in both non-irradiated and UVB-irradiated keratinocytes treated with the microalgal lipid extract. Additionally, the elevation in alkylacylPE (PEo) and diacylPE (PE) species content observed in UVB-irradiated keratinocytes following treatment with the microalgal extract suggested the potential induction of pro-survival mechanisms through autophagy in these cells. Conversely, a noteworthy reduction in LPC content in UVB-irradiated keratinocytes treated with the extract, indicated the anti-inflammatory properties of the lipid extract obtained from microalgae. However, to fully comprehend the observed alterations in the phospholipid profile of UVB-irradiated keratinocytes, further investigations are warranted to identify the specific fraction of compounds responsible for the activity of the *Nannochloropsis oceanica* extract.

## Introduction

The epidermis functions as the body’s primary defence barrier against various environmental factors, notably ultraviolet (UV) radiation, particularly UVB radiation^1^. UVB radiation disrupts the metabolism of skin cells, particularly epidermal cells^2,3^, resulting in increased production of reactive oxygen species (ROS) and alterations in the antioxidant system, mainly within keratinocytes, which are the most abundant epidermal cells^4^. These changes give rise to oxidative stress, leading to structural and metabolic modifications in vital cellular components, including phospholipids, the primary constituents of the cell membrane^5^.

Studies have reported that UVB radiation induces alterations in the composition and metabolism of phospholipids in keratinocytes^6^. Phospholipids not only serve as key structural elements of cell membranes but also act as precursors to signalling molecules and lipid mediators^7^. Oxidative stress leads to the peroxidation of polyunsaturated fatty acids in phospholipids^8^, resulting in the generation of diversified phospholipid oxidation products, including reactive unsaturated aldehydes as products of their oxidation that may have proinflammatory and deleterious effects in cells^9,10^. Concurrently, the enzymatic metabolism of phospholipids intensifies, leading to an increased production of lipid mediators, crucial for signal transduction and inflammatory processes^11^.

The overproduction of ROS is closely associated with pathological conditions in the skin, and abnormal phospholipid metabolism may contribute to the development of various skin diseases^12–15^, including skin cancer^16^. Unfortunately, the mechanisms responsible for the pathology of skin diseases have not been fully elucidated thus far. Furthermore, therapeutic approaches relying on synthetic compounds often entail significant side effects^17,18^. Therefore, there is a current scientific trend to explore exogenous and natural compounds possessing antioxidative and anti-inflammatory properties that may serve as supportive substances for the therapy of skin diseases resulting from harmful UV radiation effects.

Marine plants, such as macroalgae or microalgae, have gained increasing attention due to their rich composition of compounds with antioxidant and anti-inflammatory properties. These components from algae are being considered potential modulators of skin cell metabolism, particularly under oxidative stress conditions^19^. Algae offer a diverse array of bioactive compounds, including lipids, polysaccharides, carotenoids, vitamins, phenolics, and phycobiliproteins^20^. Algae oils are particularly enriched with polyunsaturated omega-6 and omega-3 fatty acids (PUFAs), including docosahexaenoic acid (DHA), which finds application in skin protection formulations^21^. As a result, algae biomass and extracts, especially lipid extracts, are under evaluation for their therapeutic potential in various skin conditions^22,23^, including thalassotherapy^18,24^. Different species of algae have different qualitative and quantitative composition, both in terms of lipid components as well as antioxidants^25^. Moreover, results of recent studies suggest that marine algae, particularly *Nannochloropsis oceanica*, especially its lipid extracts, may modulate the metabolism of polyunsaturated fatty acids^26^. However despite of these investigations, to the best of our knowledge, the capacity of lipid extracts from microalgae, specifically *Nannochloropsis oceanica*, to modulate lipid metabolism and membrane phospholipid composition in UV radiation-altered skin cells remains unexplored. Since no data have been published on this topic so far, in the present study we aimed to investigate the effects of lipid extracts from *Nannochloropsis oc*eanica on the phospholipid profile of human keratinocytes exposed to chronic UVB radiation.

## Materials and methods

### Microalgal lipid extracts

Lipid extraction of *Nannochloropsis oceanica* was performed using a modified Folch method as described previously^27,28^. Briefly, lipids were extracted using a solvent mixture of dichloromethane:methanol (2:1, v/v) that was added to 25 mg of biomass. The samples were vortexed and centrifuged at 670×*g* for 10 min. The supernatant was then collected, and this process was repeated three more times. The combined supernatants were dried under a stream of nitrogen. The extracts were then re-dissolved in dichloromethane and methanol, vortexed well and Mili-Q water was added. Phase separation was attained after centrifugation (670×*g* for 10 min) and the organic phase was collected. The aqueous phase was re-extracted two more times. The lipid extract was obtained by combining the organic phases. The lipid content was determined gravimetrically. The lipid profile of the obtained *Nannochloropsis oceanica* extracts was characterized by hydrophilic interaction liquid chromatography coupled with high-resolution mass spectrometry (HILIC-MS) and tandem MS (MS/MS) using a Q-Exactive hybrid quadrupole Orbitrap mass spectrometer (Thermo Fisher Scientific, Bremen, Germany) as reported previously^27^. Relative amounts of lipid classes identified in the obtained extract are given in the Table 1. The detailed composition of the algal lipid extract is given in Supplementary Table S4.

**Table 1:** Composition of *Nannochloropsis oceanica* lipid extract.

Lipid class	Relative amount (%)
Phosphatidylcholine	19.2
Lysophosphatidylcholine	8.7
Phosphatidylethanolamine	12.2
Lysophosphatidylethanolamine	5.7
Phosphatidylglycerol	6.1
Lysophosphatidylglycerol	0.4
Phosphatidylinositol	4.8
Monoacylglyceryl 3-O-4′-(*N*,*N*,*N*-trimethyl) homoserine	7.4
Diacylglyceryl 3-O-4′-(*N*,*N*,*N*-trimethyl) homoserine	14.0
Sulfoquinovosyldiacylglycerol	4.4
Monogalactosylmonoacylglycerol	5.7
Digalactosyldiacylglycerol	7.4
Monogalactosylmonoacylglycerol	0.4
Digalactosylmonoacylglycerol	1.8
Ceramides	1.7

### Cell culture

Human immortalized keratinocytes CDD 1102 KERTr (CRL2310), obtained from the American Type Culture Collection ATCC^®^ (Manassas, VA, USA), were used in the experiments. Cells from passage 8 were cultured in a humid atmosphere of 5% CO_2_ and a temperature of 37 °C. The growth medium was prepared as follows: keratinocyte-SFM medium supplemented with 1% bovine pituitary extract (BPE) and antibiotics: 50 U/ml penicillin and 50 μg/ml streptomycin. All experiments were performed under sterile conditions, including sterile plastics and cell culture reagents purchased from Gibco (Grand Island, NY, USA).

The keratinocytes, after reaching 70% confluency, were exposed to UVB radiation. The radiation dose was 60 mJ/cm^2^ (312 nm, power density at 4.08 mW/cm^2^) (Bio-Link Crosslinker BLX 312/365, Vilber Lourmat, Germany), which corresponded to approximately 70% of cell survival as measured by the MTT (3-(4,5-dimethylthiazol-2-yl)-2,5-diphenyltetrazolium bromide) method^29^ and, as previously shown, leads to the activation of prooxidative conditions^30^. To avoid heat stress, cells were irradiated in cold PBS (phosphate-buffered saline, 4 °C) and the distance of the plates from lamps was constantly maintained at 15 cm.

After irradiation, the cells were treated with a lipid extract from *Nannochloropsis oceanica* algae in 0.1% DMSO (dimethyl sulfoxide) at concentrations ranging from 1 µg/ml to 1 mg/ml for 24 h without washing under standard conditions. Control cells were incubated in parallel for 24 h with algal extracts under standard conditions (without irradiation) in a medium containing the lipid extracts from *Nannochloropsis oceanica* algae in 0.1% DMSO. The algal extract concentration selected for experiments (3 μg/ml) did not induce changes in cell viability compared to control cells measured by the MTT test^29^. After 24 h incubation, all cells were washed with PBS, harvested by scraping in cold PBS and, after disintegration, centrifuged, the resulting solution was used for lipid extraction.

### Lipid extraction from keratinocytes and quantification of total phospholipid content

The Bligh and Dyer method^31^ was used for total lipids extraction from cell pellets. The amount of phospholipids was quantified in each extract according to the Bartlett and Lewis method^32^. All experimental procedures concerning lipid extraction and phospholipid quantification were described in detail in previously published studies^33,34^.

### Phospholipid profiling by hydrophilic interaction liquid chromatography coupled with high-resolution tandem mass spectrometry (HILIC-MS/MS)

The HILIC-MS/MS was used for obtaining the keratinocytes phospholipid profile. UPLC system (Agilent 1290; Agilent Technologies, Santa Clara, CA, USA) coupled with a QTOF mass spectrometer (Agilent 6540; Agilent Technologies, Santa Clara, CA, USA) was used for the analysis. Chromatographic separation of phospholipids was performed on the Ascentis Si column (15 cm × 1 mm, 3 μm, Sigma-Aldrich) in gradient elution with the mixture of solvent A [ACN/MeOH/water 50:25:25 (v/v/v) with 1 mM ammonium acetate] and solvent B [ACN/MeOH 60:40 (v/v) with 1 mM ammonium acetate]. The QTOF mass spectrometer was operated in negative-ion mode (electrospray voltage, − 3000 V) with a capillary temperature of 250 °C and sheath gas flow of 13 L/min, as previously described^33^.

### Ceramide profiling by reversed-phase chromatography coupled with high-resolution tandem mass spectrometry RPLC-MS/MS analysis of ceramides

The ceremide profiles were obtained by using the same Agilent UPLC-ESI-QTOF-MS system (Agilent 1290; Agilent 6540; Agilent Technologies, Santa Clara, CA, USA), as in the case of phospholipid profiling. Ceremides were separated by RPLC on the RP C18 column (Acquity BEH Shield 2.1 × 100 mm; 1.7 μm; Waters, Milford, MA, USA) using methanol and water with 20 mM ammonium formate pH 5. The QTOF operating parameters and identification of ceramide species have been previously described in detail^35^.

### Data processing

Data processing including filtering, peak detection, alignment, integration and the assignment of each phospholipid and ceramide species was performed with MZmine 2.30 software based on an in-house lipid database^36^. Phospholipid and ceramide species were confirmed by mass accuracy typically less than 5 ppm (Supplementary Tables S1 and S2).

### Statistical analysis

Metaboanalyst version 5.0^37^ was used for univariate and multivariate statistical analyses. Principal component analysis (PCA) was performed on autoscaled data obtained by MS/MS analysis. Additionally, statistically significant differences between examined groups of keratinocytes were investigated using a one-way ANOVA test with a Tukey’s post hoc test. A p < 0.05 was considered statistically significant. The heatmaps were created using "Euclidean" as the clustering distance and "Ward" as the clustering algorithm.

### Measurement of neutral sphingomyelinase (SMase) activity

The SMase activity was measured using a commercial kit from Sigma-Aldrich (no. MAK152, Sigma-Aldrich, St Louis, MO, USA) according to the kit instructions. The activity was calculated by measuring the absorbance at 655 nm, using a standard curve created for colorimetric product. The activity was and reported as mU/mg of cytosolic protein. Protein level was quantified using the Bradford method^38^. The data are expressed as average ± SD (for n = 6). The obtained data were analyzed using one way ANOVA with the with Tukey’s post hoc test used for multiple comparisons to determine the significant differences between groups. A p value < 0.05 was considered significant. Statistical analyses were GraphPad Prism 7 for Windows version 7.0.0 (GraphPad Software, San Diego, CA, USA).

## Results

The HILIC-LC–MS/MS lipidomic platform allowed the identification of phospholipid species from seven classes in the extracted lipids, specifically: phosphatidylethanolamine (PE), lyso-phosphatidylethanolamine (LPE), phosphatidylcholine (PC), lyso-phosphatidylcholine (LPC), phosphatidylserine (PS), sphingomyelin (SM), and phosphatidylinositol (PI). A total of 111 most abundant phospholipid species were identified in the keratinocytes, and their details are provided in Supplementary Table S1. For relative quantification, we relied on the peak area of each phospholipid species listed in Supplementary Table S2, along with the peak area of an internal standard corresponding to each class, following a previously described method^33^. To explore significant differences in the phospholipid profiles among the groups under examination, we employed both multivariate and univariate statistics. Our analysis involved four groups of keratinocytes: non-treated cells [control], cells treated with a lipid extract from the microalga Nannochloropsis oceanica (3 µg/ml) [Algae], cells irradiated with UVB [UVB], and cells irradiated with UVB and treated with the extract from the microalga [UVB + Algae].

**Figure 1:**
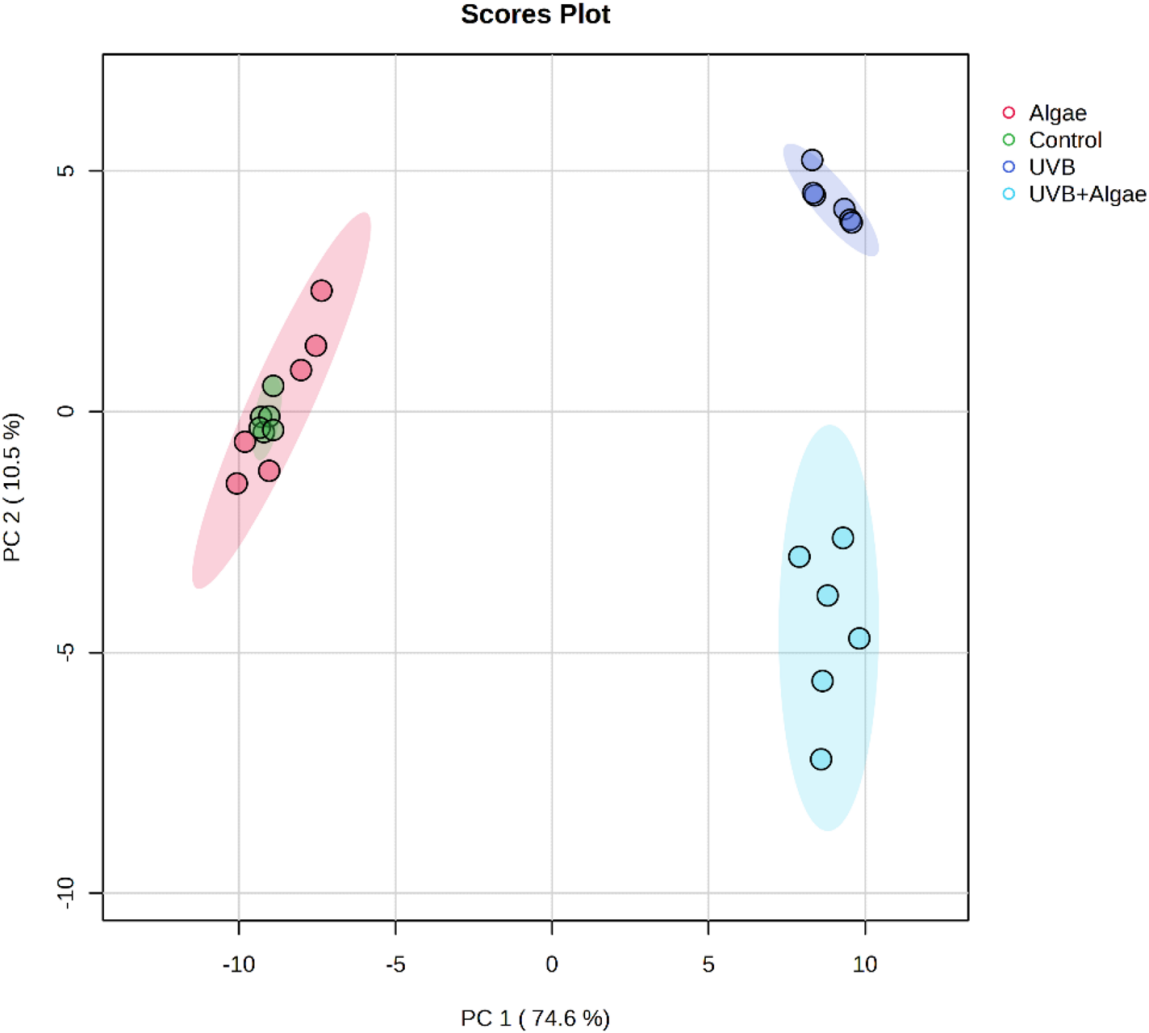
Two-dimensional principal component analysis (2D PCA) scores plot of the relative phospholipid content in non-treated keratinocytes (Control), keratinocytes treated with an extract from the microalgae *Nannochloropsis oceanica* (3 µg/ml) [Algae], keratinocytes irradiated with UVB (60 mJ/cm^2^) [UVB] and keratinocytes irradiated with UVB (60 mJ/cm^2^) and treated with an extract from the microalgae *Nannochloropsis oceanica* (3 µg/ml) [UVB + Algae].

Principal Component Analysis (PCA) of the phospholipid species in keratinocytes accounted for 85.1% of the variance (PC1: 74.6%, PC2: 10.5%) (Fig. 1). The PCA plot demonstrates that the analyzed samples clustered into three distinct groups. The PC1 component described the variation between the groups of irradiated cells (UVB and UVB + Algae) and the control group. Both groups of UVB-irradiated keratinocytes, treated and non-treated with microalga extract, was clearly separated from the groups of non-irradiated cells. This observation confirms that UVB radiation substantially altered phospholipid profile of irradiated cells. The PC2 component described the variation between the two groups of UVB-irradiated keratinocytes, those treated and those not treated with the extract from the microalgae (UVB and UVB + Algae). This indicates that phospholipid profile of UVB-irradiated keratinocytes significantly differ from UVB-irradiated cells treated with the microalga lipid extract. No discrimination was observed between the control group and the Algae group.

**Figure 2:**
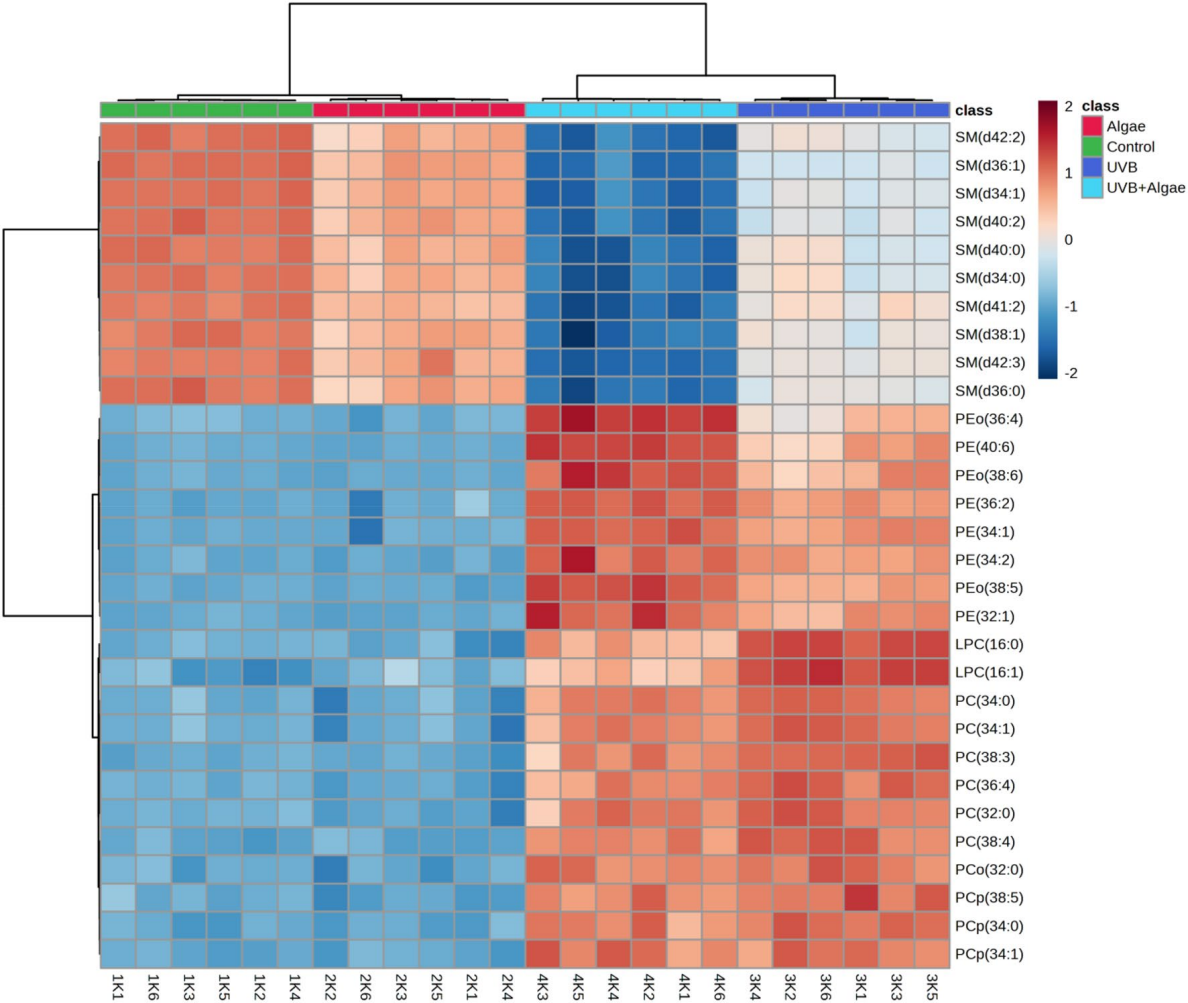
Two-dimensional hierarchical clustering heat map of the 30 main phospholipids of the four groups of keratinocytes, non-irradiated (Control), irradiated with UVB (UVB) and treated with an extract from the microalgae *Nannochloropsis oceanica* (Algae), and irradiated with UVB and treated with the extract (UVB + Algae), after the one-way ANOVA test. Levels of relative abundance are indicated on the color scale, with numbers indicating the fold difference from the grand mean. The clustering of the sample groups is represented by the dendrogram on the top. At the bottom appears the sample names. The clustering of individual phospholipid species with respect to their similarity in the change in relative abundance is shown by the dendrogram to the left.

For a comprehensive analysis of the acquired data, we conducted a one-way analysis of variance (ANOVA) and utilized Tukey's post hoc test to compare the four examined groups against each other. The dendrogram with two-dimensional hierarchical clustering in Fig. 2 illustrates the variation in relative abundance of the 30 most relevant phospholipid species (with lowest p-values) within each class under the studied conditions. The primary split observed in the upper hierarchical dendrogram demonstrates that the samples were independently clustered into four main groups. Furthermore, the individual phospholipid species, based on their similar expression changes, were clustered into two main groups. The first group predominantly comprised SM species, while the second group consisted of PC, LPC, and ether-linked PE molecular species (PEo) (Fig. 2).

### Changes in the phospholipid profile of non-irradiated and UVB-irradiated keratinocytes after treatment with extract from the microalgae *Nannochloropsis oceanica*

The results from ANOVA tests revealed that exposure to UVB radiation led to significant alterations in the phospholipid profile of irradiated keratinocytes. Specifically, exposure to UVB caused a significant up-regulation of PEo, PC, and LPC species, while the relative abundance of SM species was significantly reduced compared to that in normal keratinocytes (Fig. 2, Table 2). Furthermore, treatment of non-irradiated keratinocytes with the lipid extract did not induce significant changes in the phospholipid profile, with the exception of downregulation of SM species. However, in UVB-irradiated keratinocytes, treatment with the lipid extract, resulted in a noteworthy reduction in SM and LPC species, though to a lesser extent compared to cells treated only with UVB. This reduction was accompanied by a significant increase in PEo species content (Fig. 2, Table 2).

**Table 2:** Observed alterations in the molecular species of the 30 most discriminating (according to One-way ANOVA) phospholipid species from the PEo, PC, LPC, and SM classes in the following experimental groups: non-treated keratinocytes (Control), keratinocytes treated with an extract from the microalgae *Nannochloropsis oceanica* (3 µg/ml) (Algae), keratinocytes irradiated with UVB (60 mJ/cm^2^) [UVB], and keratinocytes irradiated with UVB (60 mJ/cm^2^) and treated with an extract from the microalgae *Nannochloropsis oceanica* (3 µg/ml) [UVB + Algae].

Keratinocytes										
PL class	Phospholipid specie	Phospholipid molecular specie	Fold-change/p value							
			Algae vs Control		UVB vs Control		UVB + Algae vs Control		UVB + Algae vs UVB	
SM	SM(d42:2)	SM(d18:1/24:1)	0.60↓	1.6E−4	1.27↓	1.3E−9	2.95↓	6.2E−11	1.67↓	3.4E−8
SM	SM(d36:1)	SM(d18:1/18:1)	0.61↓	9.5E−5	1.88↓	7.1E−13	3.69↓	4.0E−11	1.81↓	4.3E−8
SM	SM(d34:1)	SM(d18:1/16:0)	0.54↓	4.0E−5	1.58↓	6.4E−11	3.40↓	4.6E−11	1.82↓	4.1E−8
SM	SM(d40:2)	SM(d18:2/22:0)	0.55↓	1.7E−4	1.65↓	8.0E−10	3.38↓	5.7E−11	1.73↓	1.1E−7
SM	SM(d40:0)	SM(d18:0/22:0)	0.56↓	5.9E−5	1.34↓	2.1E−7	3.23↓	9.5E−11	1.89↓	2.1E−7
SM	SM(d34:0)	SM(d18:0/16:0)	0.54↓	1.6E−5	1.28↓	4.6E−7	3.17↓	1.8E−10	1.90↓	4.3E−7
SM	SM(d41:2)	SM(d18:1/23:1)	0.50↓	4.0E−7	1.20↓	2.0E−7	2.82↓	4.8E−11	1.87↓	1.6E−8
SM	SM(d38:1)	SM(d18:1/20:0)	0.54↓	4.3E−4	1.33↓	1.5E−8	3.29↓	1.7E−9	1.96↓	3.4E−7
SM	SM(d42:3)	SM(d18:2/24:1)	0.39↓	4.8E−3	1.21↓	1.3E−9	3.14↓	8.7E−14	1.94↓	8.2E−12
SM	SM(d36:0)	SM(d18:0/18:0)	0.60 ↓	5.6E−4	1.39↓	5.9E−10	3.24↓	2.7E−11	1.84↓	6.8E−9
PE	PEo(36:4)	PE(O-16:0/20:4)	ns	ns	1.94↑	3.0E−6	3.79↑	5.9E−12	1.83↑	5.2E−6
PE	PE(40:6)	PE(18:0/22:6)	ns	ns	1.70↑	3.2E−7	2.56↑	4.4E−14	0.85↑	1.2E−4
PE	PEo(38:6)	PE(O-16:0/22:6)	ns	ns	1.66↑	1.3E−7	2.34↑	3.8E−10	0.68↑	1.2E−3
PE	PE(36:2)	PE(18:0/18:2)	ns	ns	1.24↑	3.0E−12	1.51↑	8.4E−14	0.27↑	2.0E−5
PE	PE(34:1)	PE(16:0/18:1)	ns	ns	1.53↑	5.2E−11	1.84↑	1.1E−13	0.30↑	3.8E−4
PE	PE(34:2)	PE(16:0/18:2)	ns	ns	1.56↑	6.2E−12	1.95↑	3.2E−9	0.38↑	4.5E−3
PE	PEo(38:5)	PE(O-18:0/20:5)	ns	ns	1.73↑	4.7E−12	2.37↑	3.6E−12	0.63↑	4.7E−6
PE	PE(32:1)	PE(16:0/16:1)	ns	ns	1.83↑	2.3E−9	2.38↑	4.3E−9	0.55↑	5.5E−3
LPC	LPC(16:0)	LPC(16:0)	ns	ns	5.91↑	2.5E−13	4.18↑	1.0E−8	1.73↓	2.2E−5
LPC	LPC(16:1)	LPC(16:1)	ns	ns	6.28↑	6.2E−10	4.04↑	1.9E−7	2.24↓	1.4E−6
PC	PC(34:0)	PC(16:0/18:0)	ns	ns	1.68↑	3.1E−11	1.54↑	1.4E−9	ns	ns
PC	PC(34:1)	PC(16:0/18:1)	ns	ns	1.51↑	1.6E−11	1.34↑	3.8E−9	ns	ns
PC	PC(38:3)	PC(18:0/20:3)	ns	ns	1.69↑	1.5E−13	1.43↑	6.1E−8	ns	ns
PC	PC(36:4)	PC(16:0/20:4)	ns	ns	1.26↑	3.8E−11	1.07↑	3.2E−9	ns	ns
PC	PC(32:0)	PC(16:0/16:0)	ns	ns	1.80↑	1.2E−10	1.54↑	5.9E−8	ns	ns
PC	PC(38:4)	PC(16:0/22:4)	ns	ns	2.02↑	4.3E−10	1.81↑	1.0E−10	ns	ns
PC	PCo(32:0)	PC(O-16:0/16:0)	ns	ns	1.60↑	7.6E−10	1.54↑	4.1E−10	ns	ns
PC	PCp(38:5)	PC(p18:0/20:5)	ns	ns	1.61↑	3.2E−9	1.45↑	1.7E−9	ns	ns
PC	PCp(34:0)	PC(p16:0/18:0)	ns	ns	1.57↑	4.9E−11	1.44↑	7.5E−9	ns	ns
PC	PCp(34:1)	PC(p16:0/18:1)	ns	ns	1.48↑	9.4E−10	1.52↑	2.2E−9	ns	ns

### Changes in ceramide content in non-irradiated and UVB-irradiated keratinocytes after treatment with extract from the microalgae *Nannochloropsis oceanica*

Based on the results obtained from ceramide profiling, our focus was directed towards ceramides containing non-hydroxy fatty acids and sphingosine (CER[NS]) and ceramides containing non-hydroxy fatty acids and dihydrosphingosine (CER[NDS]). These two classes of ceramides were found to be the most abundant and represented the primary ceramide species among all the identified ceramides in the examined groups of keratinocytes (Supplementary Table S3).

**Figure 3:**
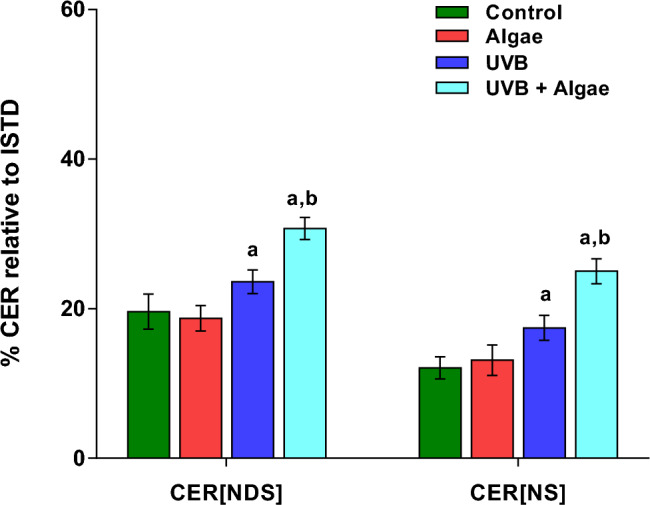
Changes in relative ceramide content within the CER[NDS] and CER[NS] classes in the following keratinocyte groups: non-irradiated (Control), irradiated with UVB (UVB) and treated with an extract from the microalgae *Nannochloropsis oceanica* (Algae), and irradiated with UVB and treated with the extract (UVB + Algae). The presented values are expressed as mean ± SD; statistically significant differences (p < 0.05) in comparison to the: a—control group, b—UVB group.

**Figure 4:**
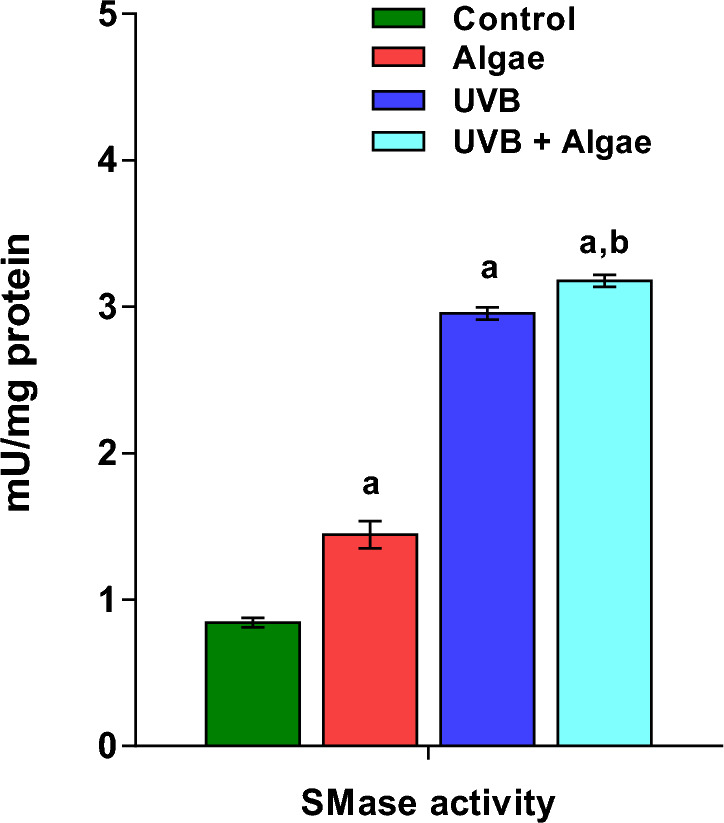
Neutral sphingomyelinase (SMase) activity in the non-irradiated keratinocytes (Control), irradiated with UVB (UVB) and treated with an extract from the microalgae *Nannochloropsis oceanica* (Algae), and irradiated with UVB and treated with the extract (UVB + Algae). The presented values are expressed as mean ± SD; statistically significant differences (p < 0.05) in comparison to the: a—control group, b—UVB group.

Our findings indicated that there were no significant changes in the relative content of both CER[NDS] and CER[NS] in control keratinocytes treated with the extract from microalgae. However, when cells were irradiated with UVB, there was a significant increase in the levels of CERs. Additionally, our results demonstrated a significant upregulation of CERs in UVB-irradiated keratinocytes that were subjected to treatment with the lipid extract from microalgae (Fig. 3). The observed increase in CER content in UVB-irradiated keratinocytes was found to be accompanied by a dramatic rise in the activity of neutral sphingomyelinase (SMase) (Fig. 4). Furthermore, our results indicated an additional significant increase in SMase activity in UVB-irradiated keratinocytes treated with an extract from microalgae, but in less extent when compared with treatment with UVB alone. Interestingly, we also observed a significantly elevated activity of SMase in non-irradiated keratinocytes treated with the microalgae extract.

**Figure 5:**
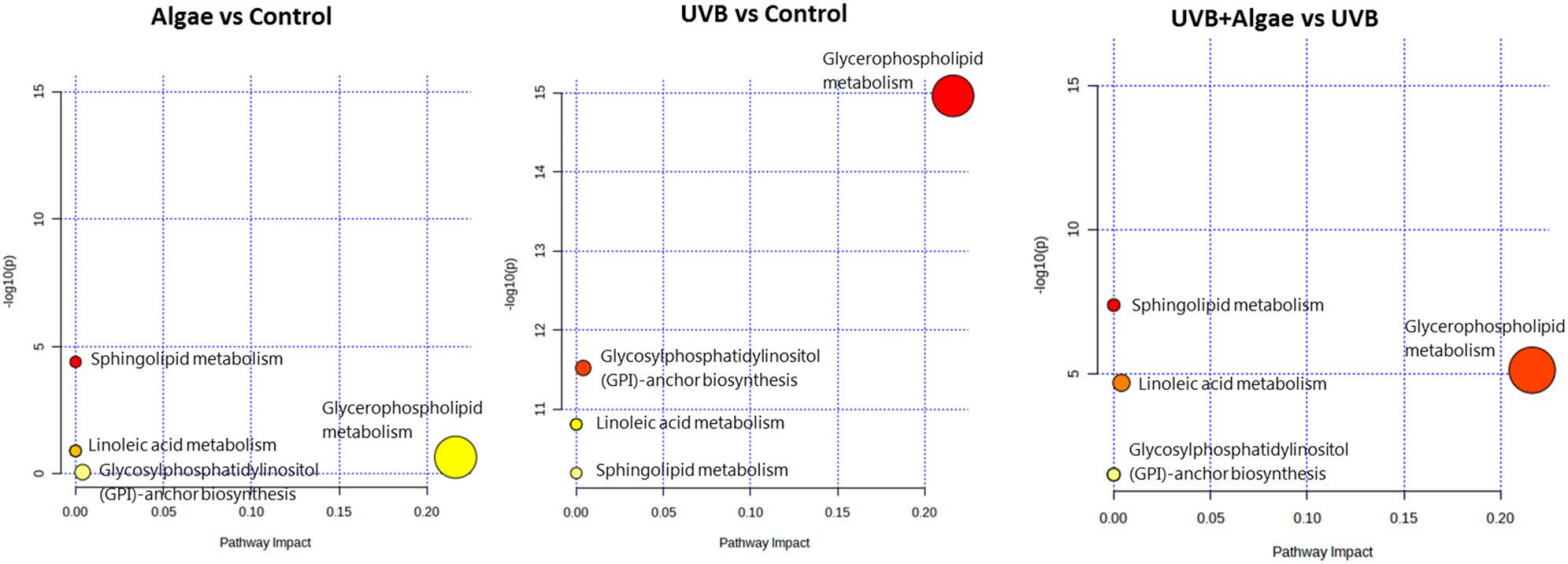
Metabolic pathway analysis performed for the 30 most discriminating (according to One-way ANOVA) phospholipid species identified in keratinocytes treated with an extract from the microalgae *Nannochloropsis oceanica* (Algae) compared to non-treated keratinocytes (Control), and UVB-irradiated keratinocytes treated with extract from the microalgae *Nannochloropsis oceanica* (UVB + Algae) compared to cells irradiated with UVB (UVB). The colour of each circle is based on the *p*-value, while the size of the circle indicates pathway impact (a combination of the centrality and number of phospholipid species enriched in the pathway). Smaller p-values and larger pathway impact circles indicate a greater perturbation of the pathway. The y-axis represents the *p*-values from pathway enrichment analysis, while the x-axis represents pathway impact values from pathway topology analysis.

We conducted a metabolic pathway analysis using MetaboAnalyst 5.0^37^ to gain an overview of the metabolic pathways associated with the identified significant phospholipids related to the effects of microalgae in keratinocytes (Fig. 5; Table 2).

The summary of metabolic pathways, which include phospholipid species discriminating the non-treated groups of keratinocytes (Control, UVB) and cells treated with the extract from the microalgae *Nannochloropsis oceanica* (Algae, UVB + Algae), is presented in Fig. 5. Among the 30 most discriminating phospholipid species (One-way ANOVA) identified in the analyzed groups of keratinocytes, a total of 7 were assigned to 5 major metabolic pathways, namely sphingolipid, glycerophospholipid, glycosylphosphatidylinositol (GPI)-anchor, as well as arachidonic and linoleic acid metabolism (Table 3). The table provides information on the number of metabolites present in each pathway and detected in keratinocytes, as well as the results of the pathway analysis (p-value and pathway impact value). Figure 5 reveals that sphingolipid metabolism was the most significantly affected metabolic pathway in non-irradiated keratinocytes treated with the lipid extract from the microalgae *Nannochloropsis oceanica*. This observation is consistent with the p-value in Table 3 and aligns with the results presented in Table 2, which showed that changes in SM content were the only effects observed upon treatment of control keratinocytes with the lipid extract from *Nannochloropsis oceanica*. In contrast, in UVB-irradiated cells, glycerophospholipid metabolism emerged as the most significantly affected metabolic pathway, while sphingolipid metabolism was found to be the least altered (Fig. 5). However, interestingly, similar to non-irradiated keratinocytes, in the case of UVB-irradiated keratinocytes treated with the lipid extract from microalgae, sphingolipid metabolism remained the most significantly affected pathway. Nevertheless, upon considering the obtained p-values (Table 3), it becomes evident that the impact of the microalgal lipid extract on cellular metabolism was more pronounced in UVB-irradiated keratinocytes, as the mentioned metabolic pathways were more affected in these cells.

**Table 3:** Metabolic pathways corresponding to phospholipid species identified in keratinocytes treated with an extract from microalgae *Nannochloropsis oceanica* (Algae) compared to nontreated keratinocytes (Control) and UVB-irradiated keratinocytes treated with an extract from microalgae *Nannochloropsis oceanica* (UVB + Algae) compared to cells irradiated with UVB (UVB).

Batch	Pathway	No. of metabolites in the pathway	No. of phospholipid species detected in keratinocytes	p-value	− log(p)	Pathway Impact
Algae vs Contol	Sphingolipid metabolism	21	1	4.0077E−5	4.3971	0.0
	Arachidonic acid metabolism	36	1	0.12447	0.90494	0.0
	Linoleic acid metabolism	5	1	0.12447	0.90494	0.0
	Glycerophospholipid metabolism	36	3	0.2204	0.65679	0.21631
	Glycosylphosphatidylinositol (GPI)-anchor biosynthesis	14	1	0.84775	0.07173	0.00399
UVB vs Contol	Glycerophospholipid metabolism	36	3	1.0928E−15	14.961	0.21631
	Glycosylphosphatidylinositol (GPI)-anchor biosynthesis	14	1	3.0137E−12	11.521	0.00399
	Arachidonic acid metabolism	36	1	1.5574E−11	10.808	0.0
	Linoleic acid metabolism	5	1	1.5574E−11	10.808	0.0
	Sphingolipid metabolism	21	1	6.4315E−11	10.192	0.0
UVB + Algae vs UVB	Sphingolipid metabolism	21	1	4.1537E−8	7.3816	0.0
	Glycerophospholipid metabolism	36	3	7.4459E−6	5.1281	0.21631
	Glycosylphosphatidylinositol (GPI)-anchor biosynthesis	14	1	2.0482E−5	4.6886	0.00399
	Arachidonic acid metabolism	36	1	0.030884	1.5103	0.0
	Linoleic acid metabolism	5	1	0.030884	1.5103	0.0

## Discussion

Phospholipids and ceramides are the predominant lipid fractions present in skin cells. They serve not only as structural components of the cell membrane, determining its permeability, but also play a crucial role in cell signaling. Consequently, the effect of external physicochemical factors on the skin may alter the metabolic response of cells. One such exogenous factor inducing oxidative stress and inflammation in skin cells is UV radiation, which in turn disturbs the metabolism of phospholipids and ceramides, leading to changes in their content and composition^39,40^.

Due to the fact that UVB is the radiation with the highest dose of energy reaching human skin and absorbed by epidermal cells, this type of radiation was selected for the experiment in relation to epidermal keratinocytes. The results obtained in this study confirm that exposure of keratinocytes to UVB radiation causes significant changes in the phospholipid profile, specifically an upregulation of PC and LPC species in UVB-irradiated keratinocytes, which aligns with findings from previous studies^6^. It has been shown that LPC may be generated by non-enzymatically, as a result of spontaneous deacylation of oxidatively-truncated phosphophatidylcholines (oxPCs), products of free radical-induced oxidation of polyunsaturated PCs^41^. However, it should be noted that LPCs are formed primarily as a result of PC hydrolysis by phospholipase A2 (PLA2) at the sn-2 position, leading to the release of fatty acids, such as arachidonic acid, a precursor of pro-inflammatory lipid mediators^42^. Given that elevated PLA2 activity was previously observed in human UVA/UVB-irradiated keratinocytes cultured in vitro^43,44^, the significant increase in LPC content observed in UVB-irradiated keratinocytes is associated with the inflammatory process induced by UV radiation. Furthermore, the observed upregulation of PC species may be related to increased PC synthesis in response to the heightened PLA2-catalyzed hydrolysis of these phospholipid species.

The results also indicate that keratinocytes exposed to UVB radiation exhibited significantly elevated relative content of ether-linked phosphatidyl-ethanolamines (PEo) species. This increase in phosphatidylethanolamines has been previously associated with up-regulation of autophagy as part of a survival mechanism^45^. Hence, it can be suggested that despite the harmful effects of UVB radiation on cellular metabolism, adaptive cellular mechanisms, including autophagy, are also induced in response to UVB radiation.

Additionally, besides the changes in the phospholipid profile of UVB-irradiated keratinocytes, there was a significant reduction in the relative content of SM species compared to non-irradiated cells. Importantly, this down-regulation of SM species in UVB-irradiated cells was accompanied by an increase in CER[NS] and CER[NDS] content, consistent with other studies demonstrating CER upregulation in UVB-exposed keratinocytes^6,46^. Several studies have shown the stimulation of CER synthesis by UVB radiation^47–49^; however, the precise mechanism of their biosynthesis remains unclear. Nevertheless, it has been previously revealed that oxidative stress resulting from UVB radiation increases the expression of acid and neutral sphingomyelinases at the mRNA level^50^. An increase in the activity of neutral and acidic sphingomyelinases has also been confirmed in human keratinocytes following exposure to UVB radiation^6^. The results obtained in this study further support these findings, as the increased CER content in UVB-irradiated keratinocytes was accompanied by a dramatic increase in neutral sphingomyelinase (SMase) activity. This suggests that increased CER synthesis in UVB-irradiated keratinocytes is a result of the degradation of sphingomyelin by SMase, one of the main pathways leading to the formation of ceramides^51,52^.

It is well-known that changes in phospholipid metabolism under UVB radiation are a consequence of shifting the redox balance in skin cells towards oxidative conditions^40,53^. Therefore, to counteract the observed changes, protective compounds with antioxidant abilities could be employed. Natural sources of antioxidants, such as microalgae, including *Chlorella vulgaris*, *Chlorococcum amblystomatis*, *Nannochloropsis gaditana* and *Nannochloropsis oceanica*, have been extensively studied for their composition and biological activity^19,26,54^. However, there have been no reports on the effect of algal extract on phospholipids in skin cells, including their metabolism. Nevertheless, recent data^26,27^ indicate the ability of microalgae *Nannochloropsis oceanica* to modulate phospholipid metabolism. Hence, in this study, we examined the effect of *Nannochloropsis oceanica* lipid extract on the phospholipid profile of non-irradiated and UVB-irradiated human keratinocytes.

The obtained results demonstrate that treatment of non-irradiated keratinocytes with the lipid extract from algae leads to a down-regulation of SM species. Notably, this was the only effect observed in this study upon exposing non-irradiated keratinocytes to the algal extract. Furthermore, the reduction in SM content was much more pronounced in UVB-irradiated keratinocytes subjected to treatment with the microalgal extract. These findings align with the results of the metabolic pathway analysis, which revealed that sphingomyelin metabolism was the most significantly affected metabolic pathway in both non-irradiated and UVB-irradiated keratinocytes after treatment with the lipid extract from microalgae *Nannochloropsis oceanica*.

Moreover, the observed down-regulation of SM in UVB-irradiated keratinocytes treated with the microalgal extract was accompanied by a significant upregulation of both classes of ceramides, CER[NDS] and CER[NS], while no significant changes in the relative content of ceramides were found in control keratinocytes treated with the lipid extract from microalgae. Although, to date, no data on the effect of microalgae *Nannochloropsis oceanica* on sphingomyelin metabolism and ceramide synthesis have been reported, our results show a significant increase in SMase activity in both non-irradiated and UVB-irradiated keratinocytes treated with the extract from microalgae. This observation clearly indicates the ability of the lipid extract from *Nannochloropsis oceanica* to modulate the SM-CER pathway. Considering that increased activity of neutral and acidic sphingomyelinases has been reported in human keratinocytes exposed to UVB radiation^6^, it is highly likely that the observed enhanced synthesis of ceramides in UVB-irradiated keratinocytes treated with the extract from microalgae is associated with further activation of sphingomyelinases. However, a clear explanation of the mechanism leading to the observed changes in the content of SM and ceramides requires further research, including the identification of the specific compounds present in the lipid extract that are involved in these mechanisms.

Another important finding of our study is the significant increase in the amount of ether-linked forms of phosphatidylethanolamine (PEo) in keratinocytes treated with the microalgal extract after UVB irradiation. Given that an increase in phosphatidylethanolamine species promotes autophagy, it can be assumed that the microalgal extract induces pro-survival mechanisms in keratinocytes to protect against UV radiation and prevent early cell death by maintaining cellular homeostasis. Notably, microalgae *Nannochloropsis oceanica* is a rich source of lutein^55^, which has been shown to induce autophagy by upregulating autophagy-related genes, such as Beclin-1 (BECN1)^56^. Additionally, ether phospholipids, including PEo, are involved in various metabolic processes as they serve as precursors of inflammatory lipid mediators/modulators^57,58^. Moreover, due to their ability to remove reactive oxygen species, ether phospholipids possess antioxidant properties^59,60^. Therefore, the increased amount of PEo species in UVB-irradiated keratinocytes observed in this study suggests that the *Nannochloropsis oceanica* lipid extract may enhance the antioxidant potential of these cells, which was also indicated in our recent paper^61^.

In our study, we observed that PE species containing linoleic acid (LA, 18:2), namely PE(18:0/18:2) and PE(16:0/18:2), were significantly up-regulated in UVB-irradiated keratinocytes, and this up-regulation was further enhanced after treatment with the microalgal extract. The metabolic pathway analysis also revealed that LA metabolism was one of the more affected metabolic pathways in both UVB-irradiated and non-irradiated keratinocytes treated with the lipid extract from microalgae *Nannochloropsis oceanica*. It is well known that the rate of oxidation of unsaturated fatty acids by ROS increases with the increase in number of double bonds^62^. Thus, phospholipids with a larger number of double bonds, e.g. with an arachidonoyl or docosahexaenoyl residue, are more susceptible to oxidation. Given that LA is highly susceptible to peroxidation among polyunsaturated fatty acids (PUFAs), it is likely that the observed up-regulation of these PE species may result from their increased synthesis in response to enhanced oxidative modifications in irradiated cells. However, the additional increase in PE(18:0/18:2) and PE(16:0/18:2) content in UVB-irradiated keratinocytes treated with the microalgal extract may be associated with the protective action of antioxidant compounds present in *Nannochloropsis oceanica* against peroxidation of PUFAs. Notably, microalgae *Nannochloropsis oceanica* has been shown to contain numerous compounds with antioxidant activity, including proteins, vitamins, and carotenoids^55,63^. Among these, carotenoids pigments such as astaxanthin, canthaxanthin, neoxanthin, violaxanthin, and zeaxanthin are largely responsible for the natural antioxidant potential of this microalga species^64^. Astaxanthin, in particular, has been shown to significantly prevent phospholipid peroxidation in human erythrocytes^65^, possibly by reducing the activity of pro-oxidative enzymes like xanthine oxidase and NADPH oxidase^66^. Additionally, astaxanthin exhibits a significant antioxidant effect on irradiated skin fibroblasts by upregulating the expression of classical antioxidant enzymes through Nrf2 activation^67,68^.

Moreover, our results demonstrate that introducing the lipid extract from *Nannochloropsis oceanica* into the medium of keratinocytes after UVB irradiation leads to a significant down-regulation of lyso-phosphatidylcholine (LPC) species, partially preventing the up-regulation induced by UVB irradiation alone. This observation may be related to the ability of compounds present in the lipid extract from microalga to regulate the activity of phospholipase A_2_ (PLA_2_). Lutein, one of the most abundant compounds in the carotenoid fraction of *Nannochloropsis oceanica*, has been shown to act as a competitive inhibitor of cytosolic PLA_2_^69^. Additionally, vitamin E, a major lipophilic antioxidant also present in the lipid extract from *Nannochloropsis oceanica*, has been found to be an excellent inhibitor of PLA_2_^70,71^. LPC elevation has been associated with increased inflammation through the activation of neutrophil NADPH oxidase^72^. Therefore, the decrease in LPC content observed in UVB-irradiated keratinocytes in our study may indicate the anti-inflammatory effect of the lipid extract from *Nannochloropsis oceanica*. Furthermore, our results support previously published data showing that microalgae species, including *Nannochloropsis oceanica*, have the ability to inhibit the production of pro-inflammatory cytokines and down-regulate the expression of inflammatory genes^19,73–76^.

## Conclusions

In summary, our study demonstrates a partially protective effect of the lipid extract from *Nannochloropsis oceanica* microalga on the phospholipids and sphingolipids, of both control and mostly of UVB-irradiated keratinocytes. The observed reductions in PEo and PE levels, along with the modulation of the SM-CER pathway, indicate potential antioxidant and anti-inflammatory properties of the microalga extract. Additionally, the significant decrease in LPC content in UVB-irradiated keratinocytes, that seem to be counteracted by the algal extracts further supports its anti-inflammatory potential. The changes in metabolic pathways involving relevant phospholipids also suggest altered cellular metabolism under the influence of the microalga extract.

However, it is important to acknowledge that this study is the first to describe the effects of the lipid extract from *Nannochloropsis oceanica* on the phospholipid profile of UVB-irradiated keratinocytes, and not all observed changes have clear interpretations. Therefore, further research is needed to elucidate the specific mechanisms underlying the observed alterations in the phospholipid profile of UVB-irradiated keratinocytes and to identify the specific compounds or groups of compounds from the *Nannochloropsis oceanica* extract that are involved in these mechanisms. Such additional investigations will provide a deeper understanding of the potential therapeutic applications of this microalga extract in skin health and protection against oxidative stress and inflammation caused by UV radiation.

### Supplementary Information

Supplementary Tables.

Supplementary Table S2.

## Data Availability

The datasets generated during and/or analysed during the current study are available online as supplementary material.
