# Supplementary material for: Lipidomic assessment of the impact of Nannochloropsis oceanica microalga lipid extract on human skin keratinocytes exposed to chronic UVB radiation
Source: Sci Rep. 2023 Dec 15;13:22302. doi: 10.1038/s41598-023-49827-2 (PMC10724133; doi:10.1038/s41598-023-49827-2)
Supplement: Supplementary file 1 — Supplementary Tables. [file 41598_2023_49827_MOESM1_ESM.docx]

**Table S1.** Most abundant phospholipid species identified in non-treated keratinocytes (Control), keratinocytes treated with extract from microalga *Nannochloropsis oceanica* (3 µg/ml) (Algae), keratinocytes irradiated with UVB (60 mJ/cm2) [UVB] and keratinocytes irradiated with UVB (60 mJ/cm2) and treated with extract from microalga *Nannochloropsis oceanica* (3 µg/ml) [UVB+Algae].

| **Phospholipid class** | **m/z** | **Retention time** | **Phospholipid specie** |
| --- | --- | --- | --- |
| **PC** | 818.5922 | 9.91 | PC(34:1) |
|  | 844.6033 | 9.76 | PC(36:2) |
|  | 790.5554 | 10.05 | PC(32:1) |
|  | 764.5424 | 10.22 | PC(30:0) |
|  | 792.5694 | 10.08 | PC(32:0) |
|  | 816.5754 | 9.89 | PC(34:2) |
|  | 846.6232 | 9.75 | PC(36:1) |
|  | 820.6021 | 9.92 | PC(34:0) |
|  | 874.6543 | 10.36 | PC(38:1) |
|  | 866.5864 | 9.52 | PC(38:5) |
|  | 804.6067 | 10.08 | PCp(34:0) |
|  | 842.5852 | 9.72 | PC(36:3) |
|  | 864.5724 | 9.53 | PC(38:6) |
|  | 778.5942 | 10.22 | PCo(32:0) |
|  | 840.5698 | 9.62 | PC(36:4) |
|  | 872.6401 | 9.65 | PC(38:2) |
|  | 776.5793 | 10.10 | PCp(32:0) |
|  | 890.5907 | 9.41 | PC(40:7) |
|  | 820.5491 | 9.73 | PCp(36:6) |
|  | 868.6026 | 9.49 | PC(38:4) |
|  | 794.5355 | 9.93 | PCp(34:5) |
|  | 788.5433 | 10.05 | PC(32:2) |
|  | 892.6051 | 9.41 | PC(40:6) |
|  | 750.5609 | 10.36 | PCo(30:0) |
|  | 870.6208 | 9.61 | PC(38:3) |
|  | 776.5436 | 10.11 | PC(34:0) |
|  | 900.6055 | 9.92 | PCp(42:8) |
|  | 850.5925 | 9.55 | PCp(38:5) |
|  | 802.5966 | 9.87 | PCp(34:1) |
|  | 838.5572 | 9.62 | PC(36:5) |
|  | 894.6252 | 9.46 | PC(40:5) |
|  | 846.5662 | 10.08 | PCp(38:7) |
|  | 872.5809 | 10.02 | PCp(40:8) |
|  | 926.6274 | 9.72 | PCp(44:9) |
|  | 824.5782 | 9.59 | PCp(36:4) |
|  | 796.5464 | 9.98 | PCp(34:4) |
|  | 748.5464 | 10.34 | PCp(30:0) |
| **LPC** | 580.3623 | 12.32 | LPC(18:1) |
|  | 554.3443 | 12.60 | LPC(16:0) |
|  | 552.3261 | 12.58 | LPC(16:1) |
| **SM** | 761.5769 | 11.24 | SM(d34:1) |
|  | 871.6866 | 10.71 | SM(d42:2) |
|  | 763.5916 | 10.96 | SM(d34:0) |
|  | 845.6737 | 10.78 | SM(d40:1) |
|  | 759.5642 | 11.26 | SM(d34:2) |
|  | 843.6546 | 10.82 | SM(d40:2) |
|  | 789.6081 | 11.08 | SM(d36:1) |
|  | 787.5971 | 10.57 | SM(d36:2) |
|  | 869.6763 | 10.69 | SM(d42:3) |
|  | 817.6399 | 10.87 | SM(d38:1) |
|  | 847.6891 | 10.31 | SM(d40:0) |
|  | 857.6755 | 11.15 | SM(d41:2) |
|  | 791.6234 | 10.49 | SM(d36:0) |
| **LPE** | 478.2959 | 6.37 | LPE(18:1) |
|  | 480.3116 | 6.33 | LPE(18:0) |
| **PE** | 742.5386 | 4.46 | PE(36:2) |
|  | 716.5256 | 4.57 | PE(34:1) |
|  | 738.5108 | 4.23 | PE(36:4) |
|  | 744.5567 | 4.44 | PE(36:1) |
|  | 714.5087 | 4.21 | PE(34:2) |
|  | 700.5262 | 4.54 | PEo(34:2) |
|  | 722.5097 | 4.33 | PEo(36:5) |
|  | 746.5112 | 4.26 | PEo(38:7) |
|  | 766.5336 | 4.26 | PE(38:4) |
|  | 748.5255 | 4.29 | PEo(38:6) |
|  | 790.5388 | 4.18 | PE(40:6) |
|  | 774.5381 | 4.15 | PEo(40:7) |
|  | 750.5424 | 4.24 | PEo(38:5) |
|  | 688.4942 | 4.68 | PE(32:1) |
|  | 726.5406 | 4.44 | PEo(36:3) |
|  | 764.5225 | 4.28 | PE(38:5) |
|  | 772.5268 | 4.17 | PEo(40:8) |
|  | 792.5497 | 4.25 | PE(40:5) |
|  | 762.5063 | 4.24 | PE(38:6) |
|  | 674.5088 | 4.66 | PEo(32:1) |
|  | 728.5558 | 4.42 | PEo(36:2) |
|  | 672.4971 | 4.65 | PEo(32:2) |
|  | 718.5377 | 4.58 | PE(34:0) |
|  | 724.5277 | 4.34 | PEo(36:4) |
| **PI** | 915.5973 | 2.06 | PI(40:3) |
|  | 863.5608 | 2.22 | PI(36:1) |
|  | 885.5498 | 2.14 | PI(38:4) |
|  | 917.6082 | 2.06 | PI(40:2) |
|  | 887.5655 | 2.15 | PI(38:3) |
|  | 835.5311 | 2.23 | PI(34:1) |
|  | 889.5769 | 2.19 | PI(38:2) |
|  | 861.5495 | 2.22 | PI(36:2) |
|  | 883.5331 | 2.15 | PI(38:5) |
|  | 913.5813 | 2.13 | PI(40:4) |
|  | 833.5154 | 2.22 | PI(34:2) |
|  | 1031.755 | 1.85 | PI(48:1) |
|  | 911.5645 | 2.13 | PI(40:5) |
|  | 837.5487 | 2.14 | PI(34:0) |
|  | 909.5433 | 2.12 | PI(40:6) |
|  | 865.5826 | 2.22 | PI(36:0) |
|  | 859.5364 | 2.12 | PI(36:3) |
|  | 891.5967 | 2.20 | PI(38:1) |
|  | 1029.737 | 1.85 | PI(48:2) |
|  | 857.5183 | 2.19 | PI(36:4) |
| **PS** | 818.5922 | 9.91 | PS(38:0) |
|  | 844.6033 | 9.76 | PS(40:1) |
|  | 790.5554 | 10.05 | PS(36:0) |
|  | 846.6232 | 9.75 | PS(40:0) |
|  | 816.5754 | 9.89 | PS(38:1) |
|  | 866.5864 | 9.52 | PS(42:4) |
|  | 864.5724 | 9.53 | PS(42:5) |
|  | 842.5852 | 9.72 | PS(40:2) |
|  | 872.6401 | 9.65 | PS(42:1) |
|  | 868.6026 | 9.49 | PS(42:3) |
|  | 840.5698 | 9.62 | PS(40:3) |
|  | 890.5906 | 9.41 | PS(44:6) |

**Table S2**. Peak area of each phospholipid species identified in non-treated keratinocytes (Control), keratinocytes treated with extract from microalga *Nannochloropsis oceanica* (3 µg/ml) (Algae), keratinocytes irradiated with UVB (60 mJ/cm2) [UVB] and keratinocytes irradiated with UVB (60 mJ/cm2) and treated with extract from microalga *Nannochloropsis oceanica* (3 µg/ml) [UVB+Algae].


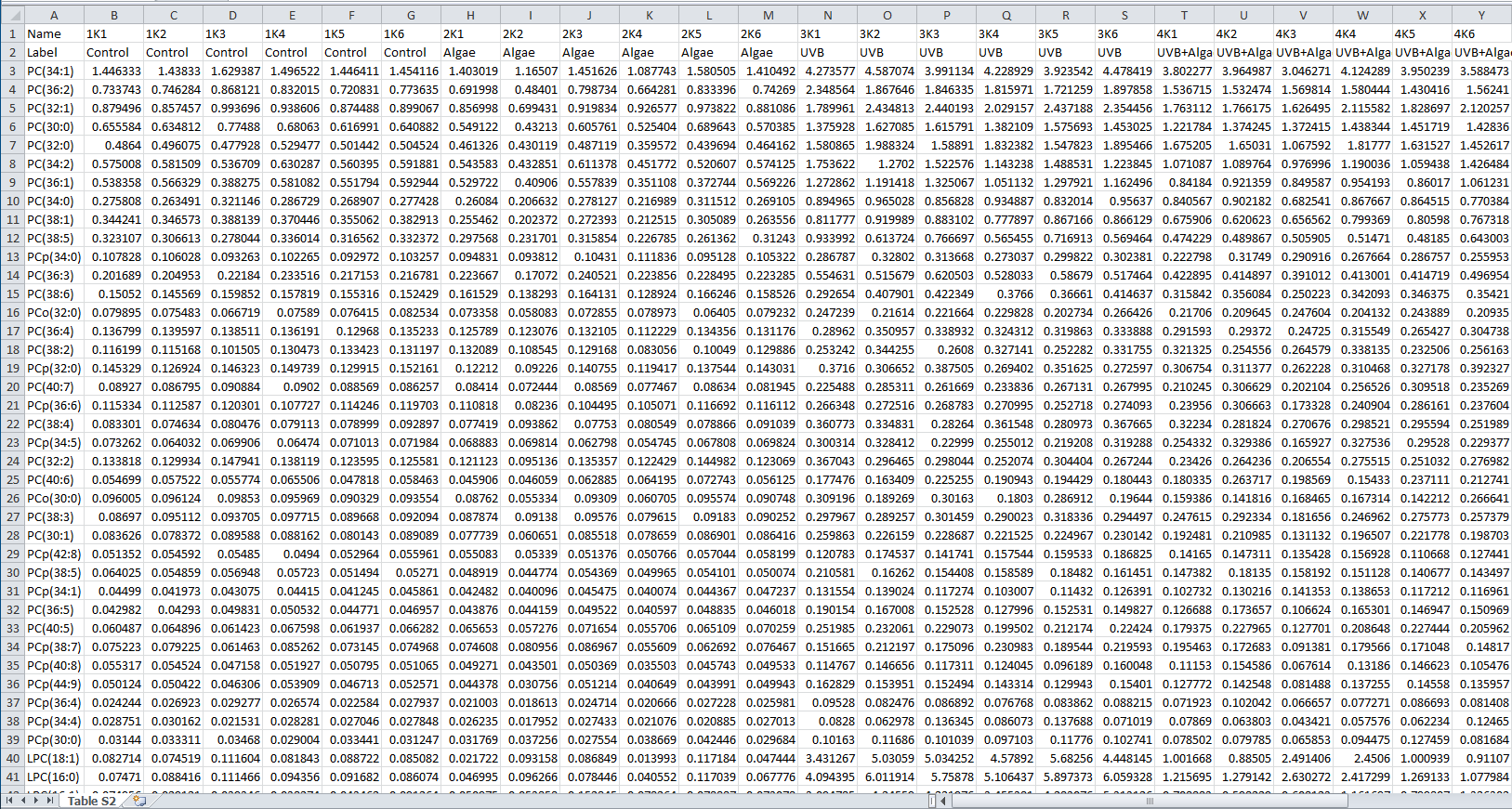


**Table S3**. Most abundant ceramide species (CER[NS], CER[NDS]) identified in non-treated keratinocytes (Control), keratinocytes treated with extract from microalga *Nannochloropsis oceanica* (3 µg/ml) (Algae), keratinocytes irradiated with UVB (60 mJ/cm2) [UVB] and keratinocytes irradiated with UVB (60 mJ/cm2) and treated with extract from microalga *Nannochloropsis oceanica* (3 µg/ml) [UVB+Algae]*.*

| **Ceramide class** | **m/z** | **Retention time** | **Ceramide specie** |
| --- | --- | --- | --- |
| CER[NS] | 536.4954 | 42.94 | Cer(d18:2/16:0) |
|  | 538.5112 | 44.27 | Cer(d18:1/16:0) |
|  | 550.5141 | 42.47 | Cer(d15:2/20:0) |
|  | 562.5127 | 44.07 | Cer(d18:2/18:1) |
|  | 564.5269 | 41.97 | Cer(d18:1/18:1) |
|  | 564.5318 | 35.12 | Cer(d16:2/20:0) |
|  | 566.5417 | 44.41 | Cer(d18:1/18:0) |
|  | 590.5441 | 41.48 | Cer(d18:2/20:1) |
|  | 592.5573 | 44.6 | Cer(d18:2/20:0) |
|  | 594.5751 | 40.07 | Cer(d18:1/20:0) |
|  | 620.5892 | 37.97 | Cer(d18:2/22:0) |
|  | 622.6081 | 36.91 | Cer(d18:1/22:0) |
|  | 648.6224 | 41.83 | Cer(d18:1/24:1) |
|  | 650.6377 | 41.81 | Cer(d18:1/24:0) |
|  | 676.6523 | 43.62 | Cer(d18:1/26:1) |
|  | 678.6656 | 43.31 | Cer(d18:1/26:0) |
| CER[NDS] | 540.5242 | 29.39 | Cer(d18:0/16:0) |
|  | 566.5417 | 33.83 | Cer(d18:0/18:1) |
|  | 568.5581 | 33.84 | Cer(d18:0/18:0) |
|  | 596.5908 | 31.78 | Cer(d18:0/20:0) |
|  | 624.6231 | 34.3 | Cer(d18:0/22:0) |
|  | 652.6532 | 33.48 | Cer(d18:0/24:0) |
|  | 678.6623 | 24.85 | Cer(d18:0/26:1) |
|  | 650.6379 | 33.36 | Cer(d18:0/24:1) |

**Table S4.** Polar lipid composition of the lipid extracts of *Nannochloropsis oceanica* identified by HILIC-ESI-MS and HILIC-ESI-MS/MS in positive ion mode, as [M+H]^+^ and [M+NH_4_]^+^ ions, and in negative ion mode, as [M-H]-. When the fatty acyl chain combination was not possible to assign the lines were filled with "-".

| **Lipid species (C:N)** | **Calculated *m/z*** | **Fatty acyl chains (C:N)** |
| --- | --- | --- |
|  |  |  |
| **PC identified as [M+H]^+^** | | |
| PC(28:1) | 676,4917 | - |
| PC(30:3) | 700,4917 | - |
| PC(30:1) | 704,523 | 16:1-14:0 |
| PC(31:2) | 716,523 | 16:1-15:1 |
| PC(31:1) | 718,5387 | 16:1-15:0 |
|  |  | 17:1-14:0 |
|  |  | 15:1-16:0 |
| PC(32:5) | 724,4917 | - |
| PC(32:4) | 726,5074 | 16:3-16:1 |
|  |  | 16:2/16:2 |
| PC(32:3) | 728,523 | 16:2-16:1 |
|  |  | 16:3-16:0 |
|  |  | 18:3-14:0 |
| PC(32:2) | 730,5387 | 16:1/16:1 |
|  |  | 16:2-16:0 |
|  |  | 18:2-14:0 |
| PC(32:1) | 732,5543 | - |
| PC(33:3) | 742,5387 | 17:2-16:1 |
|  |  | 17:1-16:2 |
|  |  | 15:0-18:3 |
| PC(33:2) | 744,5543 | 18:2-15:0 |
|  |  | 17:1-16:1 |
|  |  | 17:2-16:0 |
| PC(34:7) | 748,4917 | - |
| PC(34:6) | 750,5074 | 16:1-18:5 |
| PC(34:5) | 752,523 | 18:3-16:2 |
|  |  | 18:4-16:1 |
|  |  | 14:0-20:5 |
|  |  | 18:2-16:3 |
| PC(34:4) | 754,5387 | 20:4-14:0 |
|  |  | 16:1-18:3 |
|  |  | 18:4-16:0 |
|  |  | 18:2-16:2 |
|  |  | 18:1-16:3 |
| **Lipid species (C:N)** | **Calculated m/z** | **Fatty acyl chains (C:N)** |
| **PC identified as [M+H]^+^** | | |
| PC(34:3) | 756,5543 | 18:2-16:1 |
| PC(34:2) | 758,57 | 18:1-16:1 |
|  |  | 18:2-16:0 |
| PC(34:1) | 760,5856 | 18:1-16:0 |
|  |  | 16:1-18:0 |
| PC(35:4) | 768,5543 | - |
| PC(35:3) | 770,57 | - |
| PC(35:2) | 772,5856 | - |
| PC(35:1) | 774,6013 | 16:1-17:0 |
| PC(37:7) | 790,5387 | 17:2-20:5 |
| PC(37:6) | 792,5543 | 21:5-16:1 |
|  |  | 17:1-20:5 |
|  |  | 17:2-20:4 |
| PC(36:8) | 774,5074 | - |
| PC(36:7) | 776,523 | 16:2-20:5 |
| PC(36:6) | 778,5387 | 16:1-20:5 |
|  |  | 16:2-20:4 |
| PC(36:5) | 780,5543 | 16:1-20:4 |
|  |  | 16:0-20:5 |
| PC(36:4) | 782,57 | 18:2-18:2 |
|  |  | 20:3-16:1 |
|  |  | 20:4-16:0 |
| PC(36:3) | 784,5856 | 20:3-16:0 |
|  |  | 16:1-20:2 |
|  |  | 18:2-18:1 |
| PC(36:2) | 786,6013 | 18:1-18:1 |
|  |  | 20:2-16:0 |
|  |  | 18:2-18:0 |
|  |  | 20:1-16:1 |
| PC(38:8) | 802,5387 | 18:3-20:5 |
| PC(38:7) | 804,5543 | 18:2-20:5 |
|  |  | 18:3-20:4 |
| **Lipid species (C:N)** | **Calculated m/z** | **Fatty acyl chains (C:N)** |
| **PC identified as [M+H]^+^** | | |
| PC(38:6) | 806,57 | 18:2-20:4 |
|  |  | 18:1-20:5 |
|  |  | 20:3-18:3 |
| PC(38:5) | 808,5856 | 20:4-18:1 |
| PC(38:3) | 812,6169 | - |
| PC(38:2) | 814,6326 | 18:2-20:0 |
| PC(38:1) | 816,6482 | - |
| PC(40:11) | 824,523 | - |
| PC(40:10) | 826,5387 | 20:5/20:5 |
| PC(40:9) | 828,5543 | 20:5-20:4 |
| PC(40:8) | 830,57 | - |
| PC(40:5) | 836,6169 | - |
| **Lipid species (C:N)** | **Calculated *m/z*** | **Fatty acyl chains (C:N)** |
|  |  |  |
| **LPC identified as [M+H]^+^** | | |
| LPC(14:0) | 468,309 | 14:0 |
| LPC(16:3) | 490,2934 | 16:3 |
| LPC(16:2) | 492,309 | 16:2 |
| LPC(16:1) | 494,3247 | 16:1 |
| LPC(16:0) | 496,3403 | 16:0 |
| LPC(17:0) | 510,356 | 17:0 |
| LPC(17:1) | 508,3403 | 17:1 |
| LPC(17:2) | 506,3247 | 17:2 |
| LPC(18:5) | 514,2934 | - |
| LPC(18:4) | 516,309 | 18:4 |
| LPC(18:3) | 518,3247 | 18:3 |
| LPC(18:2) | 520,3403 | 18:2 |
| LPC(18:1) | 522,356 | 18:1 |
| LPC(20:5) | 542,3247 | 20:5 |
| LPC(20:4) | 544,3403 | 20:4 |
| LPC(20:3) | 546,356 | - |
| LPC(20:1) | 550,3873 | - |
| LPC(20:0) | 552,4029 | 20:0 |
| LPC(22:6) | 568,3403 | - |
| LPC(22:5) | 570,356 | - |
| **Lipid species (C:N)** | **Calculated *m/z*** | **Fatty acyl chains (C:N)** |
|  |  |  |
| **PE identified as [M+H]^+^** | | |
| PE(30:3) | 658,4448 | - |
| PE(30:1) | 662,4761 | 14:0-16:1 |
|  |  | 15:1-15:0 |
| PE(30:0) | 664,4917 | 14:0-16:0 |
|  |  | 15:0/15:0 |
| PE(32:4) | 684,4604 | - |
| PE(32:3) | 686,4761 | 16:2-16:1 |
| PE(32:2) | 688,4917 | 16:1/16:1 |
|  |  | 16:2-16:0 |
| PE(32:1) | 690,5074 | - |
| PE(34:6) | 708,4604 | - |
| PE(34:5) | 710,4761 | - |
| PE(34:4) | 712,4917 | 18:3-16:1 |
|  |  | 14:0-20:4 |
| PE(34:3) | 714,5074 | 18:2-16:1 |
|  |  | 20:3-14:0 |
| PE(34:2) | 716,523 | 16:1-18:1 |
| PE(36:8) | 732,4604 | - |
| PE(36:7) | 734,4761 | - |
| PE(36:6) | 736,4917 | 16:1-20:5 |
|  |  | 16:2-20:4 |
| PE(36:5) | 738,5074 | 16:1-20:4 |
| PE(36:4) | 740,523 | 20:3-16:1 |
|  |  | 20:4-16:0 |
| PE(36:3) | 742,5387 | - |
| PE(36:2) | 744,5543 | 18:1/18:1 |
| PE(38:9) | 758,4761 | - |
| PE(38:8) | 760,4917 | 18:4-20:4 |
| PE(38:7) | 762,5074 | 18:3-20:4 |
|  |  | 20:3-18:4 |
|  |  | 18:2-20:5 |
| PE(38:6) | 764,523 | 18:2-20:4 |
| PE(38:5) | 766,5387 | 18:1-20:4 |
| PE(40:10) | 784,4917 | 20:5/20:5 |
| PE(40:9) | 786,5074 | 20:4-20:5 |
| PE(40:8) | 788,523 | 20:4-20:4 |
| PE(40:7) | 790,5387 | 20:3-20:4 |
|  |  | 20:5-20:2 |
| **Lipid species (C:N)** | **Calculated *m/z*** | **Fatty acyl chains (C:N)** |
|  |  |  |
| **LPE identified as [M+H]^+^** | | |
| LPE(14:0) | 426,2621 | 14:0 |
| LPE(16:3) | 448,2464 | - |
| LPE(16:2) | 450,2621 | 16:2 |
| LPE(16:1) | 452,2777 | 16:1 |
| LPE(16:0) | 454,2934 | - |
| LPE(18:4) | 474,2621 | 18:4 |
| LPE(18:3) | 476,2777 | 18:3 |
| LPE(18:2) | 478,2934 | 18:2 |
| LPE(18:1) | 480,309 | 18:1 |
| LPE(20:5) | 500,2777 | 20:5 |
| LPE(20:4) | 502,2934 | 20:4 |
| LPE(20:3) | 504,309 | - |
| LPE(20:2) | 506,3247 | - |
| **Lipid species (C:N)** | **Calculated *m/z*** | **Fatty acyl chains (C:N)** |
|  |  |  |
| **PG identified as [M−H]^−^** | | |
| PG(30:1) | 691,455 | 14:0-16:1 |
| PG(31:0) | 707,4863 | 15:0-16:0 |
| PG(31:1) | 705,4707 | 15:0-16:1 |
|  |  | 15:1-16:0 |
| PG(32:1) | 719,4863 | 16:0-16:1 |
|  |  | 14:0-18:1 |
| PG(32:2) | 717,4707 | 16:1/16:1 |
|  |  | 16:0-16:2 |
| PG(33:1) | 733,502 | 16:1-17:0 |
| PG(34:1) | 747,5176 | 16:0-18:1 |
| PG(34:2) | 745,502 | 16:0-18:2 |
|  |  | 16:1-18:1 |
| PG(34:5) | 739,455 | 14:0-20:5 |
| PG(35:5) | 753,4707 | 15:0-20:5 |
| PG(36:2) | 773,5333 | - |
| PG(36:5) | 767,4863 | 16:0-20:5 |
|  |  | 16:1-20:4 |
| PG(36:5(OH)) | 783,4812 | (16:0-OH)-20:5 |
| PG(36:6) | 765,4707 | 20:5-16:1 |
| **Lipid species (C:N)** | **Calculated *m/z*** | **Fatty acyl chains (C:N)** |
|  |  |  |
| **LPG identified as [M−H]^−^** | | |
| LPG(16:0) | 483,2723 | 16:0 |
| **Lipid species (C:N)** | **Calculated *m/z*** | **Fatty acyl chains (C:N)** |
|  |  |  |
| **PI identified as [M−H]^−^** | | |
| PI(28:0) | 753,4554 | 14:0/14:0 |
| PI(30:1) | 779,4711 | 16:1-14:0 |
| PI(32:1) | 807,5024 | 16:1-16:0 |
|  | 807,5024 | 18:1-14:0 |
| PI(32:2) | 805,4867 | 16:1/16:1 |
|  | 805,4867 | 16:0-16:2 |
|  | 805,4867 | 18:2-14:0 |
| PI(33:1) | 821,518 | 16:1-17:0 |
|  | 821,518 | 16:0-17:1 |
| PI(34:1) | 835,5337 | 18:1-16:0 |
|  | 835,5337 | 16:1-18:0 |
| PI(34:2) | 833,518 | 18:2-16:0 |
|  | 833,518 | 16:1-18:1 |
| PI(34:3) | 831,5024 | 18:2-16:1 |
|  | 831,5024 | 16:2-18:1 |
| PI(34:5) | 827,4711 | 14:0-20:5 |
| PI(36:6) | 853,4867 | 20:5-16:1 |
| PI(40:10) | 901,4867 | 20:5/20:5 |
| **Lipid species (C:N)** | **Calculated *m/z*** | **Fatty acyl chains (C:N)** |
|  |  |  |
| **MGTS identified as [M+H]^+^** | | |
| MGTS(14:1) | 444,3325 | 14:1 |
| MGTS(14:0) | 446,3482 | 14:0 |
| MGTS(15:0) | 460,3638 | 15:0 |
| MGTS(16:4) | 466,3169 | 16:4 |
| MGTS(16:3) | 468,3325 | 16:3 |
| MGTS(16:2) | 470,3482 | - |
| MGTS(16:1) | 472,3638 | 16:1 |
| MGTS(16:0) | 474,3795 | - |
| MGTS (17:1) | 486,3795 | 17:1 |
| MGTS (17:2) | 484,3638 | 17:2 |
| MGTS(18:5) | 492,3325 | 18:5 |
| MGTS(18:4) | 494,3482 | 18:4 |
| MGTS(18:3) | 496,3638 | 18:3 |
| MGTS(18:2) | 498,3795 | 18:2 |
| MGTS(18:1) | 500,3951 | 18:1 |
| MGTS(20:5) | 520,3638 | 20:5 |
| MGTS(20:4) | 522,3795 | - |
| **Lipid species (C:N)** | **Calculated *m/z*** | **Fatty acyl chains (C:N)** |
|  |  |  |
| **DGTS identified as [M+H]^+^** | | |
| DGTS(28:1) | 654,5309 | 16:1-12:0 |
|  |  | 14:1-14:0 |
| DGTS(28:0) | 656,5465 | 14:0/14:0 |
|  |  | 12:0-16:0 |
| DGTS(30:3) | 678,5309 | 16:3-.14:0 |
| DGTS(30:2) | 680,5465 | 16:2-14:0 |
|  |  | 16:1-14:1 |
| DGTS(30:1) | 682,5622 | 16:1-14:0 |
| DGTS(32:5) | 702,5309 | 20:5-12:0 |
| DGTS(32:4) | 704,5465 | 18:4-14:0 |
| DGTS(32:3) | 706,5622 | - |
| DGTS(32:2) | 708,5778 | 16:1/16:1 |
|  |  | 18:2-14:0 |
|  |  | 16:2-16:0 |
| DGTS(32:1) | 710,5935 | 16:1-16:0 |
|  |  | 18:1-14:0 |
| DGTS(33:2) | 722,5935 | 17:1-16:1 |
|  |  | 18:2-15:0 |
|  |  | 17:2-16:0 |
| DGTS(34:6) | 728,5465 | 20:5-14:1 |
| DGTS(34:5) | 730,5622 | 20:5-14:0 |
| DGTS(34:4) | 732,5778 | 20:4-14:0 |
| DGTS(34:3) | 734,5935 | 20:3-14:0 |
|  |  | 16:1-18:2 |
|  |  | 18:3-16:0 |
| DGTS(34:2) | 736,6091 | 18:2-16:0 |
|  |  | 18:1-16:1 |
|  |  | 20:2-14:0 |
| DGTS(34:1) | 738,6248 | 20:1-14:0 |
|  |  | 16:0-18:1 |
| DGTS(36:7) | 754,5622 | 16:2-20:5 |
| DGTS(36:6) | 756,5778 | 16:1-20:5 |
| DGTS(36:5) | 758,5935 | 20:5-16:0 |
| DGTS(36:4) | 760,6091 | - |
| DGTS(36:3) | 762,6248 | 20:3-16:0 |
|  |  | 20:2-16:1 |
|  |  | 18:2-18:1 |
| DGTS(36:2) | 764,6404 | - |
| DGTS (37:5) | 772,6091 | 20:5-17:0 |
|  |  | 20:4-17:1 |
| DGTS(38:10) | 776,5465 | 18:5-20:5 |
| DGTS(38:9) | 778,5622 | 18:4-20:5 |
| DGTS(38:8) | 780,5778 | - |
| **Lipid species (C:N)** | **Calculated *m/z*** | **Fatty acyl chains (C:N)** |
|  |  |  |
| **DGTS identified as [M+H]^+^** | | |
| DGTS(38:7) | 782,5935 | 20:5-18:2 |
|  |  | 18:3-20:4 |
| DGTS(38:6) | 784,6091 | - |
| DGTS(38:5) | 786,6248 | 20:4-18:1 |
|  |  | 20:5-18:0 |
| DGTS(40:10) | 804,5778 | 20:5/20:5 |
| DGTS(40:9) | 806,5935 | - |
| **Lipid species (C:N)** | **Calculated *m/z*** | **Fatty acyl chains (C:N)** |
|  |  |  |
| **SQDG identified as [M−H]^−^** | | |
| SQDG(28:0) | 737,451 | 14:0/14:0 |
| SQDG(30:0) | 765,4823 | 16:0-14:0 |
| SQDG(30:1) | 763,4666 | 16:1-14:0 |
| SQDG(31:1) | 777,4823 | 15:0-16:1 |
| SQDG(32:1) | 791,4979 | - |
| SQDG(32:2) | 789,4823 | 16:1/16:1 |
| SQDG(32:3) | 787,4666 | - |
| SQDG(34:1) | 819,5292 | 16:0-18:1 |
| SQDG(34:2) | 817,5136 | 16:0-18:2 |
| SQDG(36:5) | 839,4979 | - |
| **Lipid species (C:N)** | **Calculated m/z** | **Fatty acyl chains (C:N)** |
| **MGDG identified as [M+NH_4_]^+^** | | |
| MGDG(30:1) | 718,5464 | - |
| MGDG(32:5) | 738,5156 | - |
| MGDG(32:2) | 744,5626 | - |
| MGDG(32:1) | 746,5777 | - |
| MGDG(34:5) | 766,5469 | 14:0-20:5 |
|  |  | 16:1-18:4 |
| MGDG(34:2) | 772,5933 | - |
| MGDG(34:1) | 774,609 | - |
| MGDG(36:6) | 792,5625 | 16:1-20:5 |
| MGDG(36:5) | 794,5782 | - |
| MGDG(38:7) | 818,5782 | - |
| MGDG(40:10) | 840,5626 | - |
| MGDG(40:9) | 842,5782 | - |
| MGDG(40:8) | 844,5939 | - |
| **Lipid species (C:N)** | **Calculated *m/z*** | **Fatty acyl chains (C:N)** |
|  |  |  |
| **DGDG identified as [M+NH_4_]^+^** | | |
| DGDG(30:1) | 880,5997 | 14:0-16:1 |
| DGDG(30:0) | 882,6154 | - |
| DGDG(32:5) | 900,5684 | - |
| DGDG(32:3) | 904,5997 | - |
| DGDG(32:2) | 906,6154 | 16:1/16:1 |
|  |  | 14:0-18:2 |
|  |  | 16:0-16:2 |
| DGDG(32:1) | 908,631 | 16:0-16:1 |
|  |  | - |
| DGDG(34:5) | 928,5997 | 14:0-20:5 |
| DGDG(34:3) | 932,631 | - |
| DGDG(34:2) | 934,6467 | - |
| DGDG(34:1) | 936,6623 | - |
| DGDG(35:5) | 942,6154 | 15:0-20:5 |
| DGDG(36:7) | 952,5997 | - |
| DGDG(36:6) | 954,6154 | 16:1-20:5 |
| DGDG(36:5) | 956,631 | - |
| DGDG(38:7) | 980,631 | 18:2-20:5 |
| DGDG(38:6) | 982,6467 | - |
| DGDG(40:10) | 1002,6154 | 20:5/20:5 |
| **Lipid species (C:N)** | **Calculated *m/z*** | **Fatty acyl chains (C:N)** |
|  |  |  |
| **MGMG identified as [M+NH_4_]^+^** | | |
| MGMG(16:0) | 510,3642 | - |
| **Lipid species (C:N)** | **Calculated *m/z*** | **Fatty acyl chains (C:N)** |
|  |  |  |
| **DGMG identified as [M+NH_4_]^+^** |  |  |
| DGMG(14:0) | 644,3857 | - |
| DGMG(16:1) | 670,4014 | - |
| DGMG(16:0) | 672,417 | 16:0 |
| DGMG(20:5) | 718,4014 | - |
| **Lipid species (C:N)** | **Calculated *m/z*** | **Fatty acyl chains (C:N)** |
|  |  |  |
| **Cer identified as [M+H]^+^** |  |  |
| Cer(d32:2) | 508,473 | d18:1/14:1 |
| Cer(d32:1) | 510,4886 | - |
| **Lipid species (C:N)** | **Calculated *m/z*** | **Fatty acyl chains (C:N)** |
| **PI-Cer identified as [M−H]^−^** | | |
| PI-Cer(d18:1/14:0) | 750,4921 | d18:1/14:0 |
| PI-Cer(d18:1/14:1) | 748,4765 | d18:1/14:1 |

***Abbreviations:***

**PC**, phosphatidylcholine;

**PE**, phosphatidylethanolamine;

**PG**, phosphatidylglycerol;

**PI**, phosphatidylinositol;

**LPC**, lysophosphatidylcholine;

**LPE**, lysophosphatidylethanolamine;

**LPG**, lysophosphatidylglycerol;

**MGTS**, monoacylglyceryl 3-O-4´-(N,N,N-trimethyl) homoserine;

**DGTS**, diacylglyceryl 3-O-4´-(N,N,N-trimethyl) homoserine;

**SQDG**, sulfoquinovosyldiacylglycerol;

**MGDG**, monogalactosyldiacylglycerol;

**DGDG**, digalactosyldiacylglycerol;

**MGMG**, monogalactosylmonoacylglycerol;

**DGMG**, digalactosylmonoacylglycerol;

**Cer**, ceramide;

**PI-Cer**, inositolphosphoceramide.
